# Identification of candidates for cyclotide biosynthesis and cyclisation by expressed sequence tag analysis of *Oldenlandia affinis*

**DOI:** 10.1186/1471-2164-11-111

**Published:** 2010-02-16

**Authors:** Qiaoping Qin, Emily J McCallum, Quentin Kaas, Jan Suda, Ivana Saska, David J Craik, Joshua S Mylne

**Affiliations:** 1 The University of Queensland, Institute for Molecular Bioscience, Brisbane, Queensland, 4072, Australia; 2 School of Agriculture and Food Science, Zhejiang Forestry University, Hangzhou, Zhejiang, 311300, China; 3 Department of Botany, Faculty of Science, Charles University, Benatska 2, Prague, 128 01, Czech Republic; 4 Institute of Botany, Academy of Sciences, Pruhonice 1, 252 43, Czech Republic; 5 Current address: Institut de Biologie Moléculaire des Plantes du CNRS, Université de Strasbourg, 12 rue du Général Zimmer, 67084 Strasbourg Cedex, France; 6 Current address: Plant Immunity Research Team, RIKEN Plant Science Center, Tsurumi-ku, Yokohama, Japan

## Abstract

**Background:**

Cyclotides are a family of circular peptides that exhibit a range of biological activities, including anti-bacterial, cytotoxic, anti-HIV activities, and are proposed to function in plant defence. Their high stability has motivated their development as scaffolds for the stabilisation of peptide drugs. *Oldenlandia affinis* is a member of the Rubiaceae (coffee) family from which 18 cyclotides have been sequenced to date, but the details of their processing from precursor proteins have only begun to be elucidated. To increase the speed at which genes involved in cyclotide biosynthesis and processing are being discovered, an expressed sequence tag (EST) project was initiated to survey the transcript profile of *O. affinis* and to propose some future directions of research on in vivo protein cyclisation.

**Results:**

Using flow cytometry the holoploid genome size (1C-value) of *O. affinis *was estimated to be 4,210 - 4,284 Mbp, one of the largest genomes of the Rubiaceae family. High-quality ESTs were identified, 1,117 in total, from leaf cDNAs and assembled into 502 contigs, comprising 202 consensus sequences and 300 singletons. ESTs encoding the cyclotide precursors for kalata B1 (*Oak1*) and kalata B2 (*Oak4*) were among the 20 most abundant ESTs. In total, 31 ESTs encoded cyclotide precursors, representing a distinct commitment of 2.8% of the *O. affinis *transcriptome to cyclotide biosynthesis. The high expression levels of cyclotide precursor transcripts are consistent with the abundance of mature cyclic peptides in *O. affinis*. A new cyclotide precursor named *Oak5 *was isolated and represents the first cDNA for the bracelet class of cyclotides in *O. affinis*. Clones encoding enzymes potentially involved in processing cyclotides were also identified and include enzymes involved in oxidative folding and proteolytic processing.

**Conclusion:**

The EST library generated in this study provides a valuable resource for the study of the cyclisation of plant peptides. Further analysis of the candidates for cyclotide processing discovered in this work will increase our understanding and aid in reconstructing cyclotide production using transgenic systems and will benefit their development in pharmaceutical applications and insect-resistant crop plants.

## Background

The first cyclotide was isolated from the plant *Oldenlandia affinis*, a member of the Rubiaceae (coffee) family, which is widely distributed throughout the Congo region of Africa. Pregnant women of the Lulua tribe would drink a tea made from an infusion of *O. affinis *leaves in order to accelerate their labours. In the early 1970s, these uterotonic properties were attributed to a peptide named kalata B1 [1,2]. It was not until 20 years later that the structure of kalata B1 was described as having a backbone that is head-to-tail cyclised and possessing a disulfide knotted three-dimensional structure [3]. Kalata B1 was later shown to be produced from a larger precursor protein [4]. The name "cyclotide" was coined [5] to describe peptides with similar properties in the Rubiaceae, Violaceae and Cucurbitaceae families. With over 100 cyclotides already sequenced, a dozen structures determined and thousands more predicted from mass spectrometry profiles, cyclotides could be the largest known family of plant peptides [6].

Cyclotides are exceptionally resistant to thermal and biochemical extremes and to treatment with endoproteases [7]. It is thought that cyclotides function as defence molecules within plants, as some have been shown to have insecticidal properties [4,8,9]. This stability has motivated the development of cyclotides as scaffolds for the stabilisation and presentation of bioactive peptides in drug design [10,11]. They also have a range of potential applications in agriculture, given their insecticidal properties [8] and nematocidal activities [12-14].

*O. affinis *is a perennial herb with a woody root that grows at altitudes up to 1,500 metres above sea level [15]. Eighteen *O. affinis *cyclotides have been sequenced to date [16,17], but their processing mechanisms have only begun to be elucidated [18,19]. Cyclotides are notably absent in model plants *Arabidopsis thaliana *and *Nicotiana tabacum *(tobacco). Transgenic expression of precursor proteins for cyclotide kalata B1 in *Arabidopsis *and tobacco produces a small proportion of the correctly processed cyclic form in addition to a number of mis-processed (truncated or elongated) linear forms not usually found in *O. affinis *[18,19]. This observation suggests that plants which do not produce cyclotides still contain machinery capable of processing them. However, efficient processing might require specially adapted pathways. Alternatively, these species might contain additional pathways that degrade cyclotides or inhibit proper cyclotide processing.

Sequencing of expressed sequence tags (ESTs) obtained from plant tissues and organs has been used to provide insights into the machinery underpinning biological processes. Lange *et al. *[20] found enzymes responsible for essential oil metabolism using ESTs from a cDNA library made from *Mentha × piperita *(peppermint) leaves rich in essential oils. Davis *et al. *[21] later used ESTs to elucidate the nature of production of menthone, the predominant monoterpene produced in the essential oil of maturing peppermint. Using an EST approach, Newcomb *et al. *[22] discovered genes responsible for fruit ripening, flavour, colour and biosynthesis of health-related compounds in apple.

In this study, we estimated the genome size of *O. affinis *using flow cytometry and report an EST sequencing project for *O. affinis *that resulted in the isolation of 1,117 high-quality ESTs. This is the first significant sequencing effort of *O. affinis *and a step towards building a valuable resource that will help understand the molecular basis of cyclotide biosynthesis. We found *O. affinis *commits 2.8% of its transcriptome to cyclotide precursors and 2.4% to proteins predicted to be involved in cyclotide processing. Discovery of the proteins responsible for efficient processing of cyclic peptides will enable the efficient use of model plants or plant cells for generating cyclotides or engineered molecules with pharmaceutically active peptides grafted within the cyclic peptide backbone. These two possibilities could lead to novel crop protection strategies and the use of plants as tools for cost-effective production of peptide drugs.

## Results and Discussion

### *Oldenlandia affinis *genome size

**Figure 1:**
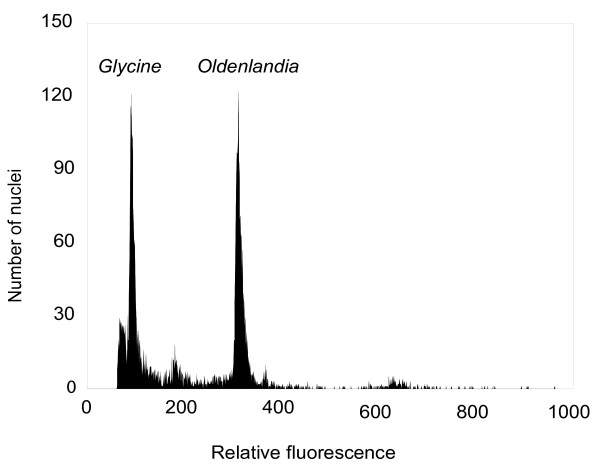
**Fluorescence histogram of propidium iodide-stained nuclei isolated from fresh leaf tissues of *Oldenlandia affinis *seedlings**. *Glycine max *'Polanka' (2C = 2.50 pg) was used as an internal reference standard. Nuclei from the sample and the standard were simultaneously isolated, stained, and analysed. The actual ratio between both peaks in this histogram is 3.42. Each plant was re-estimated three times on different days.

The somatic chromosome number of *O. affinis *is 2n = 18 (x = 9), which is in agreement with other *Oldenlandia *species, such as *O. corymbosa*, where chromosome numbers are nine or a multiple thereof [15]. The genome size of *O. affinis *was determined by using flow cytometry with *Glycine max *'Polanka' (2C = 2.50 pg) as an internal reference standard. Figure 1 shows the DNA histogram obtained from the *O. affinis *seedlings and the internal reference standard. Genome sizes (mean 2C-values ± SD) for *O. affinis *seedlings and adult plants were estimated to be 8.61 ± 0.08 pg and 8.76 ± 0.07 pg, respectively. Considering 1 pg = 0.978 × 10^9 ^bp = 978 Mbp [23], the monoploid genome size is between 4,210 and 4,284 Mbp. Certain variations between different ontogenetic stages (difference 1.7%) can be due to different levels of secondary metabolites and instrument fluctuation. 1C-values in Rubiaceae plants vary from 0.35 to 5.08 pg, with median and mean values of 0.83 pg and 1.11 pg, respectively http://data.kew.org/cvalues. *O. affinis *therefore possesses one of the largest holoploid genomes of the Rubiaceae family.

### EST sequencing and analysis

Using a cDNA library made from *O. affinis *leaves [4], we generated 1,163 ESTs. After editing raw sequences for quality and discarding sequences less than 100 bp in length, 1,117 sequences remained with an average read length of 624 bp. 60% of the ESTs were above 600 bp and 48% were above 700 bp (Table 1). All edited EST sequences were submitted to GenBank dbEST [GenBank: GE470292-GE471408]. The 1,117 ESTs were clustered into 502 contigs, comprising 202 consensus sequences and 300 singletons using SeqMan sequence assembly software (DNASTAR). Clone redundancy was 45%. Of the total ESTs, 27% represent singletons, which is similar to the values of 27% in cotton [24], 27.1% in bean [25], and 29% in pineapple [26]. To investigate the level of redundancy, contigs were listed by clone abundance, from contigs containing the largest number of ESTs to those containing a single EST. Almost half of the ESTs (48%) were found to cluster within just 16% of contigs, thus transcription of a small subset of genes appears to dominate a high proportion of the transcriptome. However, as library construction involved an amplification step, the possibility that amplification bias might have contributed to the high level of redundancy cannot be discounted.

**Table 1:** Summary of ESTs from *Oldenlandia affinis *leaf cDNA library

	Length	No. ESTs	%	Cumulative %
Minimum sequence length	105 bp			
Average sequence length	624 bp			
Maximum sequence length	926 bp			
length > 900 bp		4	0.3%	
length at 800-899 bp		165	14.8%	15.1%
length at 700-799 bp		372	33.3%	48.4%
length at 600-699 bp		136	12.2%	60.6%
length at 500-599 bp		162	14.5%	75.1%
length at 400-499 bp		119	10.7%	85.8%
length at 300-399 bp		76	6.8%	92.6%
length at 200-299 bp		48	4.3%	96.9%
length at 100-199 bp		35	3.1%	100.0%

Total No. high-quality ESTs		1,117*		

*Raw sequences were automatically trimmed using SeqMan (DNASTAR). After editing only clones above 100 bp in length were retrieved.

### Bioinformatics

To assign putative functions to *O. affinis *ESTs, sequences were exported in FASTA format and conducted with the Basic Local Alignment Search Tool (BLASTX) and performed against the GenBank non-redundant database [27,28]. A stringent BLASTX cut-off of 10E^-20 ^was chosen to ensure annotations were based on cDNAs with a high degree of similarity. Using these parameters, 21% of ESTs did not show significant homology to coding sequences in the GenBank non-redundant database and therefore could not be confidently annotated. BLASTX of the predicted proteins from the longest open reading frame of these unannotated ESTs resulted in the majority of ESTs (75%) falling below the 10E^-20 ^cut-off value chosen, with the remaining 25% not showing significant homology to any GenBank proteins. Those undiscovered sequences from *O. affinis *include novel coding sequences, as well as sequences that are non-protein coding. Due the stringent cut-off limit of 10E^-20^, ESTs with a short length of overlapping open reading frame (ORF) sequence or small ORFs were not annotated.

79% of ESTs were annotated by similarity to coding sequences in the GenBank non-redundant database. Of these, approximately 44% of ESTs shared 80% or higher identity with existing sequences, 24% had identity of between 60 - 80%, and less than 10% shared less than 60% identity to known coding sequences. Of these ESTs, 29% were predicted to be full length cDNA clones, starting with an ATG codon at a similar position to its closest BLASTX hit.

### Functional annotation

ESTs returning a valid BLASTX hit from GenBank were individually searched against The Arabidopsis Information Resource (TAIR) [29] and the Munich Information Center for Protein Sequences (MIPS) MAtDB database [30]. Annotation of *O. affinis *ESTs was based on sequence homology to *Arabidopsis *genes; ESTs were then functionally annotated according to the MIPS FunCat schema [31].

**Figure 2:**
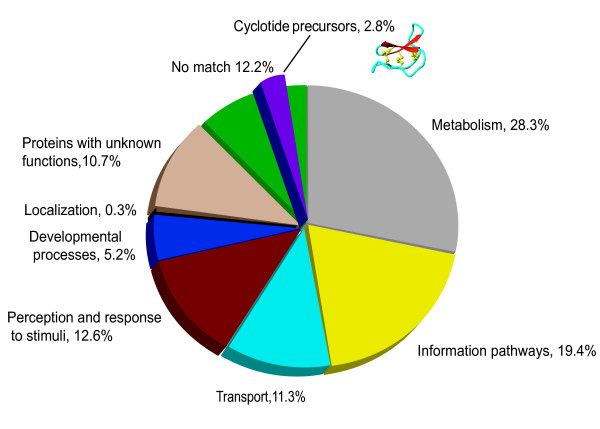
**The functional distribution of EST clones by modified MIPS based functional classification for *O. affinis***. BLASTX comparisons to predicted proteins from *Arabidopsis *were used to assign *O. affinis *ESTs based on functional annotation after MIPS FunCat schema. The cyclotide precursors are pointed out within "*No match*" group since *Arabidopsis *does not contain cyclotides.

Of the 880 ESTs returning a valid BLASTX hit, 12.2% could not be functionally annotated by comparison with genes in *Arabidopsis*. Cyclotide precursor genes fell within this group since *Arabidopsis *does not produce cyclotides. 10.7% of ESTs were found to encode proteins of unknown function. The remaining ESTs were grouped into six main categories (Figure 2): metabolism (28.3%), information pathway (19.4%), transport (11.3%), perception and response to stimuli (12.6%), developmental processes (5.2%) and localisation (0.3%). These were further subdivided into 19 subcategories (Table 2) [31].

**Table 2:** MIPS FunCat analysis of *O. affinis *ESTs compared with Arabidopsis

MIPS Functional category	No. ESTs	%
**Metabolism**		
01 metabolism	196	22.3%
02 energy	53	6.0%
**Information pathways**		
10 cell cycle and DNA processing	7	0.8%
11 transcription	9	1.0%
12 protein synthesis	60	6.8%
14 protein fate (folding, modification, destination)	88	10.0%
16 protein with binding function or cofactor requirement (structural or catalytic)	7	0.8%
**Transport**		
20 cellular transport, transport facilities and transport routes	99	11.3%
**Perception and response to stimuli**		
30 cellular communication/signal transduction mechanism	11	1.3%
32 cell rescue, defense and virulence	74	8.4%
34 interaction with the environment	25	2.8%
36 systemic interaction with the environment	1	0.1%
**Developmental processes**		
40 cell fate	13	1.5%
41 development (systemic)	16	1.8%
42 biogenesis of cellular components	9	1.0%
43 cell type differentiation	6	0.7%
45 tissue differentiation	1	0.1%
47 organ differentiation	1	0.1%
**Localisation**		
70 subcellular localisation	3	0.3%
Proteins with unknown functions	94	10.7%
No match*	107	12.2%

*The cyclotide precursors are within this subcategory.

### Contigs composed of most abundant ESTs

Analysis of EST frequency (abundance) can provide insights into gene expression levels and biochemical functions occurring in a tissue. We identified the 20 most abundantly expressed contigs (Table 3). Two contigs of these 20 most abundant ESTs were cyclotide precursor protein genes for kalata B1 (*Oak1*) and kalata B2 (*Oak4*), which is consistent with kalata B1 and B2 being the most abundant in cyclotide profiles [16,17].

**Table 3:** The 20 most abundant ESTs isolated from leaf cDNA library of *O. affinis*

Contig ID	No. ESTs	Putative function	AGI number
Oa113	21	30S ribosomal protein	AT5G24490
Oa126	18	RuBisCO activase	AT2G39730
Oa018	14	undiscovered sequence	AT3G21200
Oa122	14	mitochondrial carnitine Acyl carrier-like protein	AT5G46800
**Oa008**	**13**	**kalata B2 precursor (*Oak4*)**	
Oa014	13	CAAX protease	AT4G01320
Oa131	13	acidic endochitinase (CHIB1)	AT5G24090
Oa031	12	light-dependent NADPH:protochlorophyllide oxidoreductase B	AT4G27440
Oa054	12	fructose-bisphosphate aldolase	AT4G38970
Oa066	12	galacturonosyltransferase	AT5G54690
Oa185	12	glyceraldehyde-3-phosphate dehydrogenase	AT1G13440
**Oa064**	**11**	**kalata B1 precursor (*Oak1*)**	
Oa071	11	S-adenosylmethionine synthetase 3	AT2G36880
Oa081	11	co-chaperone grpE family protein	AT1G36390
Oa090	11	protein binding	AT1G22970
Oa188	11	asparaginase	AT3G16150
Oa134	10	ankyrin repeat family protein	AT2G01680
Oa115	9	ribosomal protein L1 family protein	AT3G63490
Oa022	8	phosphoribulokinase	AT1G32060
Oa186	8	glyceraldehyde-3-phosphate dehydrogenase	AT1G12900

*The cyclotides precursors are highlighted.

### Identification of ESTs involved in cyclotide biosynthesis

Although the number of known cyclotide peptide sequences has grown to >140 in recent years [5,32], there is still relatively little known about the mechanism by which these peptides are processed in plants. Based on previous work [18,19], we believe that cyclotide precursors enter the secretory pathway where folding and disulfide bond formation occur before cleavage and cyclisation events release the mature cyclic peptides. The presence of putative endoplasmic reticulum (ER) signal sequences in the precursors suggests that cyclotide domains probably fold in the ER where the cysteine residues form three highly conserved disulfide bonds. After correct folding and disulfide bond formation, the precursor protein is processed by cleavage of two peptide bonds and the cyclotide domain is head-to-tail cyclised. Candidate genes for these processes have been identified in this EST sequencing project.

### Cyclotide precursor proteins

**Figure 3:**
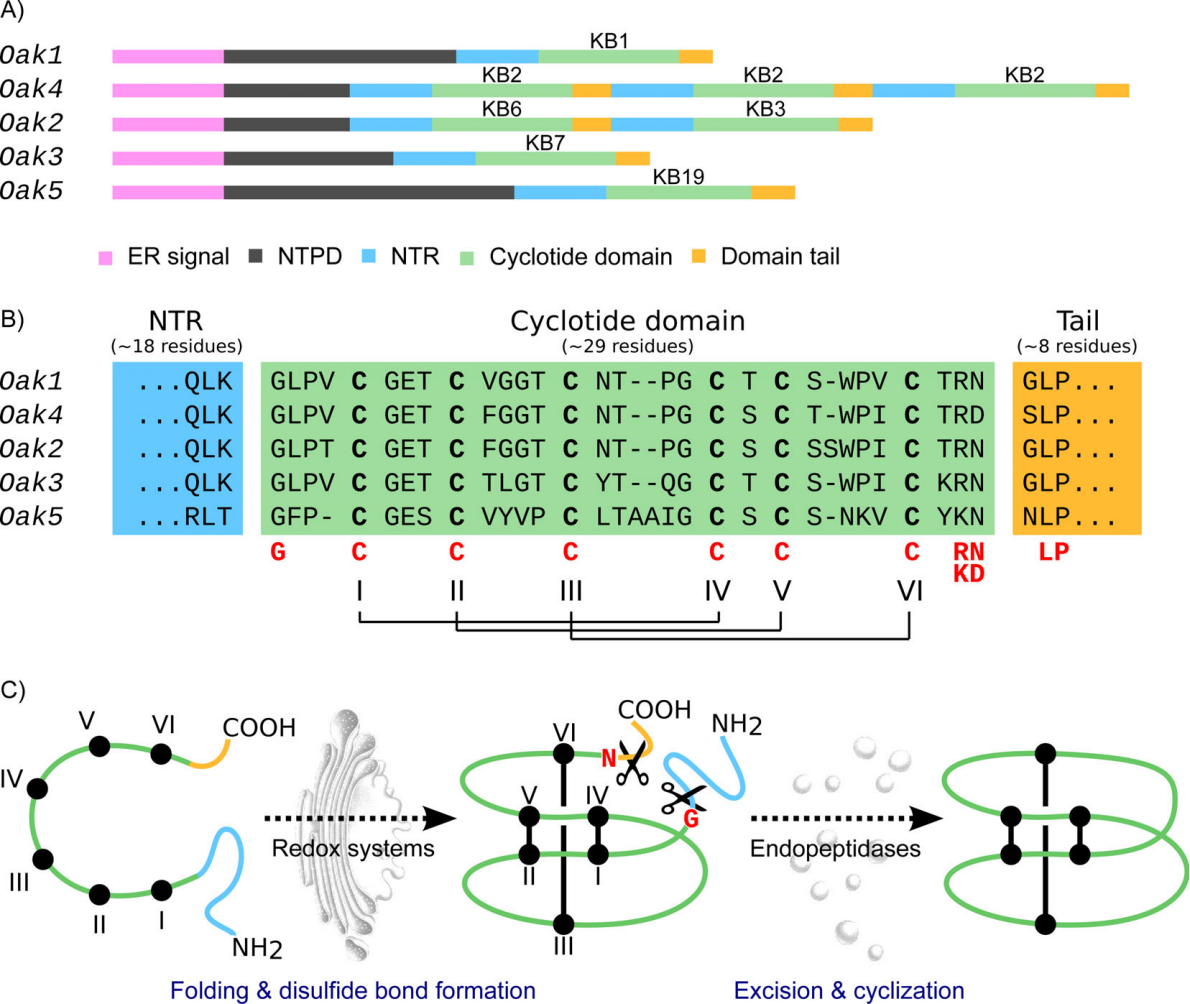
**Cyclotide precursor constructions and maturation in *O. affinis***. A) Cyclotide precursor constructions of *O. affinis*. (ER, endoplasmic reticulum; NTPD, N-terminal pro-domain; NTR, N-terminal repeat; "kB" is the abbreviation of kalatas). B) Alignment of cyclotide domains including their flanking sequences. Red letters under the sequences indicate conserved or relatively conserved amino acids. The six conserved cysteines are numbered using Roman numerals and their connectivity is shown. C) Proposed molecular model for *in vivo *formation of cyclotides in *O. affinis*. Formation of disulfide bond requires redox systems, which consists of the ferredoxin/thioredoxin system, the NADP/thioredoxin system and the glutathione/glutaredoxin system. Excision of the cyclotide domains from precursor proteins and their cyclisation are thought to be catalysed by endopeptidases [18,19]. Round black disks represent the cysteins and they are connected by disulfide bonds showing in the structures.

The cyclotide precursor proteins of *O. affinis *identified so far include an ER signal sequence, a N-terminal pro-domain of variable length and sequences, followed by one to three cyclotide domains (Figure 3) [4,33]. Usually each cyclotide domain (28 - 37 residues) is separated by a 25 amino acid region termed the N-terminal repeat (NTR) because it is relatively well conserved within these precursors. Although not part of the mature cyclotide domain, it has been suggested that NTR sequences within the precursors might modulate protein folding [33].

**Figure 4:**
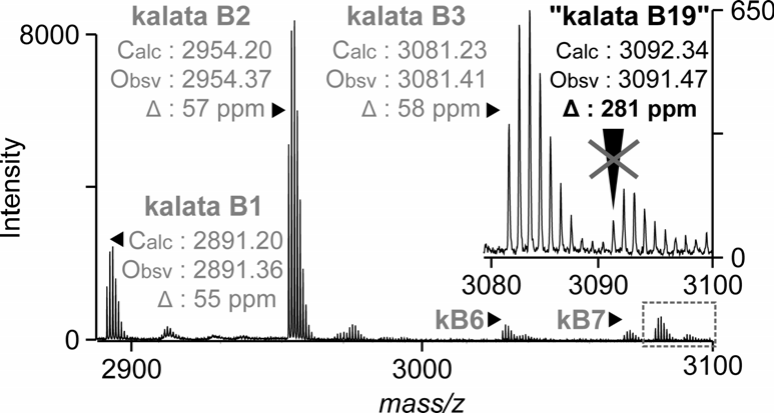
**MALDI-TOF MS profile of crude peptide extracts from *O. affinis *leaves**. The calculated (Calc) and observed (Obsv) monoisotopic masses for the cyclotides kalata B1, B2 and B3 are included and serve as internal controls. Peaks for kalata B6 (kB6) and kalata B7 (kB7) are marked without mass data. The calculated mass for kalata B19 (3092.34) is close to a mass in this profile (3091.47), but when its error (represented as ppm) is compared to the errors of other cyclotides in the same profile, the difference between calculated and observed is too great for this mass to be B19.

Four cDNA clones encoding cyclotide precursors have been isolated previously from *O. affinis*. Named *Oak1 - 4*, they encode cyclotides kalata B1, B3/B6, B7 and B2 respectively [GenBank: *Oak1 *AF393825, *Oak2 *AF393826, *Oak3 *AF393827, *Oak4 *AF393828]. We identified 13 ESTs encoding *Oak4 *(Oa008), 11 encoding *Oak1 *(Oa064), three ESTs encoding *Oak2 *(Oa119) and three ESTs encoding *Oak3 *(Oa070). MALDI-TOF mass spectrometry of leaf peptide extracts showed an abundance of these cyclotides consistent with the frequency of the corresponding ESTs (Figure 4); that is, kalata B2 and kalata B1 are most abundant while kalatas B3, B6 and B7 are less so. This result indicates that cyclotide precursor mRNA expression levels correlate with peptide levels in leaves of *O. affinis *and have not been skewed by amplification of the library by PCR.

Initially 13 ESTs were assembled into contig Oa008 encoding *Oak4*, the precursor protein for kalata B2, with 90% minimum match percentage. Re-alignment by ClustalW [34] showed that ESTs within this contig can be divided into two subgroups: namely one subgroup of nine ESTs with 100% identity to the kalata B2 precursor *Oak4 *[GenBank: AF393828] and another of four ESTs with 97% and 95% similarity to *Oak4 *at the nucleotide and protein level, respectively. The major differences were found in the 5' untranslated region and at the beginning of the ORF within the ER signal domain. They are most probably variant copies of *Oak4*, potentially produced by gene duplication.

Previous studies have employed mainly chemical peptide extractions, reverse phase-HPLC and mass spectrometry to isolate cyclotide sequences [6,35]. However, only four cDNA sequences for *O. affinis *cyclotide precursors have been determined [4] among the eighteen characterised cyclotides using the approach described above [16]. Because cyclotides precursors from *O. affinis *are only moderately conserved, with sequence identities of approximately 60%, it is difficult to develop a robust strategy for PCR amplification based on sequence similarity. In this EST project, we identified a single EST, contig Oa278, which encodes a previously unidentified putative bracelet cyclotide precursor, which we named *Oak5*. The cyclotide it encodes was named kalata B19. This is the first bracelet cyclotide precursor cDNA sequence identified in *O. affinis*; all other known cyclotide gene sequences from *O. affinis *belong to the Möbius sub-class. Möbius sub-class cyclotides contain a conceptual 'twist' (i.e. a *cis*-X-Pro peptide bond) in their backbone, whereas bracelet cyclotides do not [5]. Oa278 showed a high degree (80%) of identity with *Leonia cymosa *cycloviolin-D [Swiss-Prot: P84640] [36], *Viola hederaceae *cycloviolacin-H1 [Swiss-Prot: P58433] [5], and *Melicytus ramiflorus *Mra21, Mra14 and Mra20 [GenBank: ABO21615, ABO21616, ABO21614] [37]. The Asn residue immediately after the mature cyclotide domain in Oak5 is unusual and may affect processing. We confirmed the sequence of the *Oak5 *EST by independently cloning *Oak5 *by RT-PCR with specific primers and leaf cDNA.

The sequence of kalata B19 was compared to the characterised cyclotides from *O. affinis *and it possesses several residues that are conserved across all cyclotides (Table 4), namely six Cys residues and a Gly residue at the N-terminus. Typically, cyclotides contain a conserved Glu in loop 1 (except B12 which contains Asp), Gly in loop 3 (except Ser in B12), and Asn or Asp at the C-terminus. A proline in loop 5 is only conserved in the Möbius subfamily. This conservation still allows for the existence of a great diversity of cyclotide sequences; however it indicates that cyclotide processing for all precursors probably uses the same basic mechanism.

**Table 4:** Sequences of *O. affinis *cyclotides

**Möbius**		**amino** **acids**		**average mass (Da)**	**Ref.**
B1	G-LPVCGETCVGGTC---NTPGCTCS-WPVCTRN		29	2892.4	Saether et al. [3]
B4	G-LPVCGETCVGGTC---NTPGCTCS-WPVCTRD		29	2893.3	Craik et al. [5]
B2	G-LPVCGETCFGGTC---NTPGCSCT-WPICTRD		29	2955.4	Craik et al. [5]
B11	G-LPVCGETCFGGTC---NTPGCSCT-DPICTRD		29	2884.3	Plan et al. [16]
B15	G-LPVCGESCFGGSC---YTPGCSCT-WPICTRD		29	2976.4	Plan et al. [16]
B6	G-LPTCGETCFGGTC---NTPGCSCSSWPICTRN		30	3029.4	Jennings et al. [4]
B10	G-LPTCGETCFGGTC---NTPGCSCSSWPICTRD		30	3030.4	Plan et al. [16]
B13	G-LPVCGETCFGGTC---NTPGCACDPWPVCTRD		30	3036.5	Plan et al. [16]
B14	G-LPVCGESCFGGTC---NTPGCACDPWPVCTRD		30	3022.4	Plan et al. [16]
B3	G-LPTCGETCFGGTC---NTPGCTCDPWPICTRD		30	3082.5	Craik et al. [5]
B7	G-LPVCGETCTLGTC---YTQGCTCS-WPICKRN		29	3071.6	Jennings et al. [4]
**Bracelet**					
B8	GSVLNCGETCLLGTC---YTTGCTCNKYRVCTKD		31	3283.8	Daly et al. [55]
B9	GSVFNCGETCVLGTC---YTPGCTCNTYRVCTKD		31	3272.7	Plan et al. [16]
B12	G--SLCGDTCFVLGC---NDSSCSCN-YPICVKD		28	2880.3	Plan et al. [16]
B16	G--IPCAESCVYIPCTITALLGCKCQ-DKVCY-D		30	3186.8	Plan et al. [16]
B17	G--IPCAESCVYIPCTITALLGCKCK-DQVCY-N		30	3185.8	Plan et al. [16]
B18	G--VPCAESCVYIPC-ISTVLGCSCS-NQVCYRN		30	3145.7	Seydel et al. [17]
B5	G--TPCGESCVYIPC-ISGVIGCSCT-DKVCYLN		30	3061.6	Craik et al. [5]
B19	G--FPCGESCVYVPC-LTAAIGCSCS-NKVCYKN		30	3093.7	this work
	* *.::* * .* * :*				

Multiple sequence alignment of *O. affinis* cyclotides.

To establish whether *Oak5*-encoded kalata B19 was detectable in the *O. affinis *cyclotide profile, MALDI-TOF mass spectrometry was employed. We did not observe a mass consistent with the calculated mass of kB19 in a peptide extract from *O. affinis *leaves (Figure 3). The inability to detect kB19 is the cyclotide profile could be due to Oak5 mis-processing, but may also be because it falls below the level of detection. *Oak5 *mRNA is lowly expressed relative to the other *Oak *genes and if at low levels kB19 may be being masked by other cyclotides.

In total, 31 ESTs encoding putative cyclotide precursors were isolated. This represents 2.8% of all ESTs sequenced, and a substantial commitment of the *O. affinis *transcriptome to cyclotide biosynthesis. In *O. affinis*, cyclotides are produced in levels as high as 1 - 2 grams per kg wet weight [38], but until now it was uncertain whether this high protein expression correlated with highly abundant mRNA at the level of transcription. Cyclotides have been purified and sequenced from decades-old dried herbarium samples (D. Craik, unpublished), which indicates that cyclotides are remarkably stable in plants. The stability of cyclotides could have meant peptide levels slowly accumulated over the life of the developing plant eventually reaching high levels in adult plants, the stage at which most plants are used for studies. It is interesting to note that mRNA expression levels correlate well with peptide level, suggesting cyclotides might be subject to turnover *in planta*.

### Disulfide formation and oxidative protein folding

Previous studies show that six cysteine residues in cyclotide precursors form three disulfide bonds [39]. Disulfide bonds are required for the stability and function of a large number of proteins and disulfide bond formation is essential for proper protein folding [40]. There are several systems linking hydrogen donors to an intermediary disulfide protein, namely the ferredoxin/thioredoxin system, the NADP/thioredoxin system and the glutathione/glutaredoxin system; each system acts to effect changes that alter the activity of target proteins. These systems are composed of reduced ferredoxin, thioredoxin, reduced glutathione, glutaredoxin, ferredoxin-thioredoxin reductase, and NADP-thioredoxin reductase [41]. A related disulfide protein, protein disulfide isomerase (PDI) acts in protein assembly [42].

An *O. affinis *PDI cDNA clone [GenBank: EF611425] was isolated recently and shown to encode a protein that improves *in vitro *oxidative folding of kalata B1 at physiological pH [43]. In the current study several other ESTs involved in disulfide bond formation have been identified: Oa408 and Oa412 encoding putative thioredoxins, Oa099 and Oa312 encoding putative glutathione transferases, Oa299 encoding a putative glutaredoxin as well as Oa272 and Oa321 encoding ferredoxins. These enzymes are potential candidates for cyclotide disulfide formation and protein folding in *O. affinis*.

Thioredoxins are disulfide reductases that promote disulfide bond formation *in vivo *in the oxidised form, and glutaredoxins are disulfide bond-formation catalysts in *E. coli *[44,45]. The putative thioredoxin Oa408 has 60% sequence similarity with thioredoxin *m *from *Populus trichocarpa *[GenBank: EEF06869], wheras Oa412 has 58% similarity with thioredoxin *x *from *Arabidopsis *[GenBank: AAF15952]. Predicted protein products from both contigs contain features typical of thioredoxins; the conserved thioredoxin catalytic site motif (WCGPC), a characteristic tryptophan residue at the active thiol/disulfide site, and a structural motif thought to be involved in cell-to-cell transfer, which is typical of thioredoxins [46]. Although glutaredoxins function in a similar way to thioredoxins, glutaredoxins have a more positive redox potential [42,43]. Oa299, encoding a putative glutaredoxin family protein, shares approximately 40% sequence identity with other plant glutaredoxins [GenBank: XP_002270142, NP_196885].

A number of other ESTs encoding predicted proteins involved in the regulation of redox environment were identified and included peroxiredoxins (Oa399 and Oa400), and oxidoreductases (Oa031, Oa076, Oa465, Oa476; represented by 16 ESTs). These enzymes typically maintain the redox environment which is crucial for proper disulfide bond formation *in vivo *[41,47,48], and as such might have a role in the processing of cyclotides.

### Excision and ligation of cyclotide domain(s)

The mechanism of excision and cyclisation that occurs to produce mature cyclotides from their precursor proteins remains unclear. Excision of the cyclotide domain(s) from the precursor and ligation of the newly formed N- and C-termini probably occurs after folding of the cyclotide domain, but possibly occurs synchronously with formation of the cyclised backbone [18,19].

Although the residues preceding the cyclotide domain are not highly conserved there is a string of conserved sequence flanking the C-terminal Asn/Asp of the mature cyclotide domain. Some insight into the mechanism for carboxyl terminus processing has been gained (Figure 3) [18,19]. A residue with a small side chain (Gly, Ala, Ser) usually follows the mature cyclotide domain and itself is followed by an absolutely conserved leucine residue. Asparaginyl endopeptidases (AEPs) are widespread in plants where they are also referred to as vacuolar processing enzymes (VPEs), and specifically cleave peptide bonds on the C-terminal side of Asn and, with less efficiency, Asp [49,50]. The activity of AEP is involved in cyclisation of kalata B1 in tobacco plants transiently transformed with the *Oak1 *gene [19]. In *Oak1 *transformed tobacco plants, AEP catalyses protein backbone cyclisation by coupling asparaginyl bond hydrolysis at the C-terminus of the cyclotide domain with peptide bond ligation.

In the current study we identified an AEP homolog from *O. affinis *[GenBank: EF015631], which shares up to 68% identity with other plant AEPs [e.g. GenBank: EEF45813, P49043, BAC54827]. The putative AEP from *O. affinis *has the active site "EACES", which is conserved in plant AEPs (E(A/G)CES) [51,52]. A possible site for self-cleavage of C-terminal propeptides is present at residue 418 (VDD/W), which is conserved in most of plant AEPs [50]. This putative enzyme belongs to the vegetative-type according to genetic analysis. Since the C-terminus of cyclotide precursors *Oak1*, *Oak2*, *Oak3 *are Asn, followed by Gly, we believe AEPs have an important role in cyclotide domain excision from the precursor proteins. We are currently investigating function of AEPs on the cyclotide hydrolysis and cyclisation in *O. affinis*.

Asparaginases are enzymes that catalyse the hydrolysis of Asn to Asp, releasing NH_4_^+^, and have an important role in protein metabolism [53,54]. A putative asparaginase, Oa188 comprised of 11 ESTs, is one of the most highly expressed genes in *O. affinis *leaves according to this study (Table 3) and shares up to 86% identity with other plant asparaginases [GenBank: EEF52347, BAC66615]. There are two pairs of cyclotides in *O. affinis *that differ from each other by an Asn/Asp change at the C-terminal residue (kalata B6/B10 and kalata B1/B4). Precursor cDNAs for only one of each pair have been cloned and encode Asn-ending cyclotides, but cDNAs for their Asp-ending counterpart have not yet been identified. The abundance of cDNAs for an enzyme that catalyses a change between Asn and Asp could suggest that these cyclotides are not encoded by different gene products, rather they are produced as a result of post-translational modification.

## Conclusions

In this study we estimated the genome size of *O. affinis *using flow cytometry, and undertook an EST sequencing project on *O. affinis *to identify candidates for *in vivo *cyclotide biosynthesis and cyclisation. We discovered that the holoploid genome size of *O. affinis *was one of the largest genomes of the Rubiaceae family. 1,117 high-quality ESTs were obtained and annotated representing 502 unique transcripts. *O. affinis *dedicated a substantial portion of its transcriptome to cyclotide production: 2.8% of ESTs encoded cyclotides. We also discovered a new cDNA encoding an unidentified cyclotide kalata B19 (*Oak5*), the first cDNA for a bracelet cyclotide in *O. affinis*. As the first sequencing project undertaken in *O. affinis*, this study has uncovered ESTs encoding genes that are potentially involved at each step of cyclotide production and processing, including several cyclotide precursor proteins (*Oak1 - 5*), folding and disulfide bond formation, regulation of redox environment, and proteolytic processing. Previous attempts to express cyclotides by a transgene in model plants displayed low efficiency and mis-processing; therefore further investigation of the enzymes identified in this study could lead to a deeper understanding of cyclotide processing and improved outcomes for recombinant cyclotide production in plants, which will advance the application of cyclotides for pharmaceutical outcomes and for the protection of crop plants from insect pests.

## Methods

### Genome size determination

Genome size was measured by flow cytometry using Partec CyFlow cytometer (Partec GmbH, Münster, Germany) equipped with a 100 mW green (532 nm) solid state laser Cobolt Samba. Sample preparation followed the simplified two-step procedure described by Doležel *et al. *[23] and *Glycine max *'Polanka', 2C DNA = 2.50 pg was used as a suitable internal reference standard. Nuclei from *O. affinis *and the *Glycine max *'Polanka' were isolated simultaneously, stained with propidium iodide, and analysed. Three repetitions on different days were performed.

### cDNA library construction and EST sequencing

A leaf cDNA library was constructed [4] and amplified by PCR using universal primers M13 forward and reverse. The resulting PCR products were separated by electrophoresis and fragments larger than 500 bp recovered and cloned into pGEM-T Easy (Invitrogen) for sequencing. The Australian Genome Research Facility Ltd (Brisbane, Australia) randomly selected white colonies, prepared plasmid and sequenced each using the SK primer (5'-CGC TCT AGA ACT AGT GGA TC-3').

### EST processing and contig assembly

Raw EST sequences were automatically trimmed of vector, adapter, and low-quality sequence regions using SeqMan (Lasergene, DNASTAR Inc, Madison, USA). Clones less than 100 bp were eliminated from the data set. All edited EST sequences were submitted to GenBank dbEST. Edited sequences were assembled into contigs or singletons using SeqMan sequence assembly software with the key parameters of minimum 90% match over 50 bp overlap. Contigs were individually inspected for low-quality sequence, splice variants and chimeras, before ESTs were reassembled.

### BLAST analysis and functional annotation

Analysis of sequences was conducted with the BLASTX server and the National Center for Biotechnology Information non-redundant protein database [27,28]. The nearest neighbour hits information was parsed. Returning hits better than the 10E^-20 ^E-value cutoff value imposed were compared with proteins from TAIR [29] and searched against MAtDB to assign functional classification [30]. Functional classification was based on the MIPS FunCat schema [31]. Alignment of ESTs was conducted by Clustal W2 [34] and cyclotide precursors were compared with known sequences in the cyclotide sequence database CyBase [32].

### Confirmation of novel cyclotide precursor *Oak5*

Since *Oak5 *was represented by only a single EST, we confirmed its sequence by cloning it from *O. affinis *cDNA. *O. affinis *leaves were ground to a fine powder under liquid nitrogen and extracted by resuspension of ~0.3 mL tissue powder in 0.5 mL of buffer (0.1 M Tris pH 8.0, 5 mM EDTA, 0.1 M NaCl, 0.5% SDS, 1% 2-mercaptoethanol), extracting twice with 1:1 phenol:chloroform and precipitating the nucleic acids. Nucleic acid pellets were re-dissolved in 0.5 mL of water and the RNA selectively precipitated overnight at 4°C by addition of lithium chloride to 2 M. Total RNA was DNase treated before 1 μg was reverse transcribed using Superscript III (Invitrogen). Primers were designed against contig Oa278 which encodes Oak5. PCR was performed using primers JM302 (5'-GAG CTG GGG TGG AGC TTT T-3'), JM303 (5'-TAT TCC AAT TGG GCA ACA AG-3') and the products were cloned into pGEM-Teasy (Promega). Three clones were sequenced and confirmed the *Oak5 *sequence as correct.

### Peptide analysis by MALDI-TOF mass spectrometry

Leaf extracts were ground in 50% (v/v) acetonitrile (ACN) containing 0.05% (v/v) trifluoroacetic acid (TFA). Ground tissue was pelleted by centrifugation and the supernatant was freeze-dried and resuspended in 0.05% TFA. The C18 Ziptip (Millipore) was used to desalt and concentrate the sample. The α-cyano-4-hydroxycinnamic acid in 50% ACN/0.05% TFA was used as the matrix. Analysis of peptide masses was performed using MALDI-TOF mass spectrometer (Voyager-DE STR) (Applied Biosystems) in positive ion reflector mode with an accelerating voltage of 20,000 V. A 165 ns delay time was used. The data were acquired and processed using the accompanying software. 50 spectra at 20 positions were accumulated per spot. Calibration was conducted using a mixture of peptide standards (MSCal1; Sigma-Aldrich), and only masses above a signal to noise ratio of 5 - 10 were recorded.

## Authors' contributions

All authors were involved with experiments and data collection. DC and JM planned the project. QQ, JM, QK and EM designed and performed experiments. QQ and QK annotated the ESTs. IS performed some gene characterisation. JS performed the genome size determination. QQ, EM, DC and JM wrote the manuscript and all authors read and approved the final manuscript.
